# *Aggregatibacter actinomycetemcomitans* GroEL Protein Promotes Conversion of Human CD4+ T Cells into IFNγ IL10 Producing Tbet+ Th1 Cells

**DOI:** 10.1371/journal.pone.0049252

**Published:** 2012-11-12

**Authors:** Tahsin Saygılı, Semih Can Akıncılar, Bünyamin Akgül, Ayten Nalbant

**Affiliations:** Molecular Immunology and Gene Regulation Laboratory, Department of Molecular Biology and Genetics, Izmir Institute of Technology, Urla Izmir, Turkey; University of Iowa Carver College of Medicine, United States of America

## Abstract

One of the heat shock family protein (Hsp) expressing bacteria is the gram negative, periodontal pathogen *Aggregatibacter actinomycetemcomitans* (Aa). *A. actinomycetemcomitans*’ Hsp is a 64-kDa GroEL-protein, which has been shown to influence the host cells. In this study we used recombinant *A. actinomycetemcomitans* GroEL (rAaGroEL) protein as a model antigen to study GroEL-mediated T cell immune response. Human peripheral mononuclear cells (PBMCs), when stimulated with recombinant rAaGroEL, expressed early activation marker CD69 and IL-2R (CD25). CD25 and CD69 expressions were higher in CD4+ T cells compared to CD8+ T cells. rAaGroEL-responding CD4+ T cells expressed IL-10, IFNγ and TNFα cytokines. Interestingly, there were also IL-10 and IFNγ double cytokine producing CD4+ T cells. Additionally, IFNγ expressing CD4+ T cells were also T-bet positive. Altogether the results suggest that rAaGroEL protein affects CD4+ T cells to differentiate into IFNγ IL10-secreting T-bet+ Th1 cells.

## Introduction

Heat shock proteins (Hsps) are a group of proteins with highly conserved sequence similarity among species from bacteria to human [1]. Apart from their well-known role in protein folding, bacterial Hsp60 can modulate immune system cells to manipulate host immune response. One of the GroEL (also called Hsp60) expressing bacteria is a periodontal pathogen *A. actinomycetemcomitans* which retains its antigenic property after deleting well- characterized virulence factors [2]. This observation indicates that AaGroEL might affect human T cell function.

Bacterial Hsp60s such as AaGroEL can have a potential to modulate immune system cells. In fact there are reports in the literature that support the potential role of bacterial GroEL as an immunomodulator. For instance, in *Helicobacter pylori*-infected human patients, T cells were activated in response to *H. pylori* hsp60, to secrete IFNγ and IL-10. However, the origin of IL-10 was not reported [3]. *Brucella abortus* recombinant GroEL protein primed CD4+ T cells in vaccinated mice proliferated and the proliferating cells produce IL-2 and IFNγ in T cell culture supernatants [4]. After immunisation of BALB/c mice with *Brucella abortus* GroEL heat-hock gene, splenic T cells produced high level of IFNγ suggesting Th1 response [5]. Furthermore, recombinant hsp60 of *Salmonella typhi* immunized mice also showed higher IFNγ and IL-2 levels when splenocytes were cultured with GroEL [6]. These studies suggest that bacterial GroEL induces Th1 type immune response. However, some of these studies did not measure the cytokine expression with cell surface associated phenotypic markers. As a result, the origin of cytokines is not well characterized. Thus, It is important to unravel the T cells that specifically secret these cytokines. Furthermore, none of these studies show GroEL responding CD4+ T cells can be double cytokine producing Th1 cells.

In this study, we used recombinant AaGroEL protein as a model antigen to study bacterial Hsp-mediated CD4+ T cell immune response. To this extent, human peripheral blood mononuclear cells were cultured with rAaGroEL and cytokine profiles of CD4+ T cells were measured. Our data suggested that rAaGroEL-responding CD4+ T cells have the capacity to differentiate into IFNγ IL-10 producing Tbet+ Th1 cells. Thus, to our best knowledge, our data first time demonstrated that bacterial recombinant GroEL protein of *A. actinomycetemcomitans* polarized periferal blood CD4+ T cells into IFNγ IL-10 double cytokine producing T-bet+ Th1 cells.

## Materials and Methods

### Human Peripheral Blood Mononuclear Cells

Ethics approval for this study was obtained from the Dokuz Eylül University, İzmir, Turkey. All blood donors participated in this study were systemically and periodontally healthy adult volunteers. Subjects were asked to sign an informed consent that was previously approved by the Bioethics Committee of Dokuz Eylül University. Venous blood was drawn from the volunteers. Peripheral blood mononuclear cells (PBMC) were isolated by Ficoll-Hypaque density gradient centrifugation [7].

**Figure 1:**
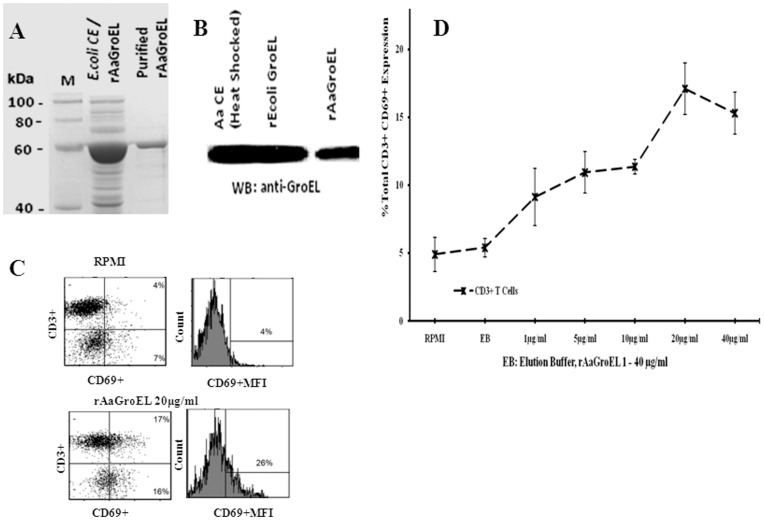
GroEL protein induces T cells activation. *(A)* Overexpression of rAaGroEL in *E.coli* cells. M: Marker, E.coliCE/rAaGroEL: rAaGroEL overexpressed E.coli lysate, and purified rAaGroEL: purified rAaGroEL protein by using Talon Resin. *(B) A. actinomycetemcomitans* sonic extract (AaCE), recombinant *E.coli* GroEL, purified rAaGroEL protein was confirmed with western blot by using anti-GroEL antibody of *E.coli*. *(C)* show representative flow data of CD69 expressing T cells. *(D)* Expression of CD69 by T cells. Human PBMCs cultured 48 h with a various rAaGroEL doses (1–40 µg/ml), RPMI and Elution Buffer (EB) which is used for rAaGroEL purification. At 48 h, cells were labeled anti-CD3 and anti-CD69 antibodies. CD3+ and non-CD3 cells were gated and analyzed for their CD69 expression. Data are representative of 3 experiments with different donors. Error bars represent standard deviation and *indicates p<0.05.

### Preparation of Recombinant AaGroEL

The genomic sequence of 64-kDa AaGroEL was first cloned into pGEM T Easy (Promega) vector [8] and then transferred into pET28a+ (Novagen) expression vector [9]. The confirmed pET/AaGroEL vector was transformed into *BL21 (DE3) E.coli* cells for protein expression. The protein purification from cell extract was carried out with TALON Cell Thru Resin according to the manufacturer’s instructions (Clontech). The purity and concentration of the eluted protein was confirmed by 8% SDS-PAGE and Bradford Protein Assay (Bio-Rad) respectively. The protein identity was further confirmed with western blotting and MS analysis. Possible LPS contamination of purified protein sample was checked by using LAL chromogenic endpoint assay (Hycult Biotechnology). Detoxi-gel endotoxin removing gel (Thermo, Fisher Scientific Inc) was used to remove LPS contamination of rAaGroEL purified samples according to manufacture instructions.

**Figure 2:**
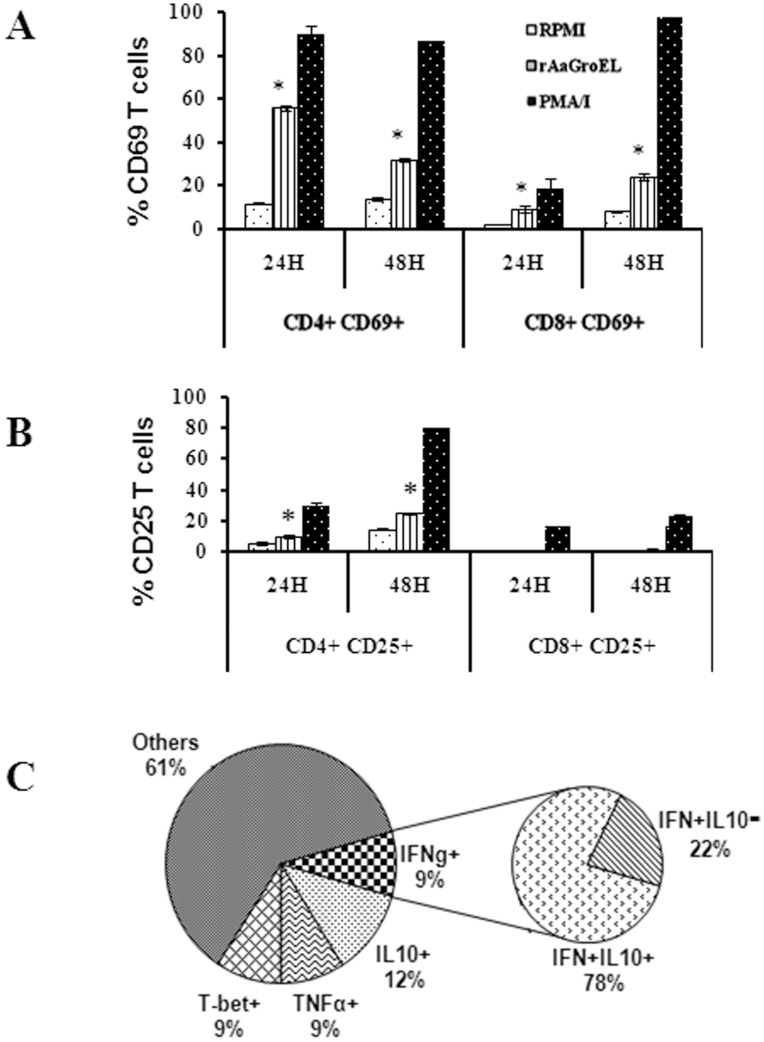
GroEL protein preferentially activates CD4+ T cells. Following 24 h and 48 h stimulation of PBMCs with rAaGroEL (20 µg/ml), cells were labeled with anti-CD3, anti-CD4, anti-CD8, anti-CD69 and anti-CD25 antibodies. CD8+ and CD4+ gated T cells were analyzed for CD69 and CD25 expressions. *(A)* CD69 expression of CD4+ and CD8+ T cells at 24 h and 48 h. *(B)* CD25 expression of CD4+ and CD8+ T cells at 24 h and 48 h. *(C)* Representative distribution of PBMCs and CD69 and CD25 expressing rAaGroEL responding CD4+ T cells. Data are representative of experiments with cells from 3 donors. Error bars represent standard deviation and *indicates p<0.05.

### Stimulation of Peripheral Mononuclear Cells

PBMCs were cultured at a concentration of 2×10^6^ cells/mL in a volume of 500 µl. Cells were incubated at different time points (2–96 h) with or without stimulants at 37°C in a humidified incubator with 5% CO_2_. The medium consisted of RPMI-1640 supplemented with 10% fetal bovine serum (FBS) (Gibco), 100 units/mL penicillin, 100 pg/mL streptomycin (Biochrom AG) and 25 mM HEPES buffer (Gibco). The purifed rAaGroEL (1–40 µg/ml), PMA (25 ng/ml) in the presence of Ionomycin (1 µg/ml) or PHA (1 µg/ml) (Biochrom AG) as positive controls and elution buffer and medium alone as negative controls were used to stimulate the cells. Each set of culture condition in all experiments was carried out as triplicates. The recombinant AaGroEL protein used in this study was expressed in *E.coli.* Therefore to address LPS contamination, we monitored TNFα expression of CD4+ T cells in different culture conditions including purified-rAaGroEL, LPS-removed-purified-rAaGroEL, purified-rAaGroEL-treated with Proteinase K and heat-inactivated-proteinase K-treated-rAaGroEL.

### Phenotypic Characterization of Cells

PBMCs were stimulated with various concentration of recombinant AaGroEL as well as negative and positive controls and were labeled with selected combination of cell surface antibodies including anti-CD3, anti-CD4, anti-CD8, anti-CD14, anti-CD25 and anti-CD69 (BD Biosciences). Cell surface labeling was carried out according to manufacturer’s instructions (BD Biosciences). Labeled cells were acquired by FACSArray and analyzed by FACSArray software or FlowJo.

**Figure 3:**
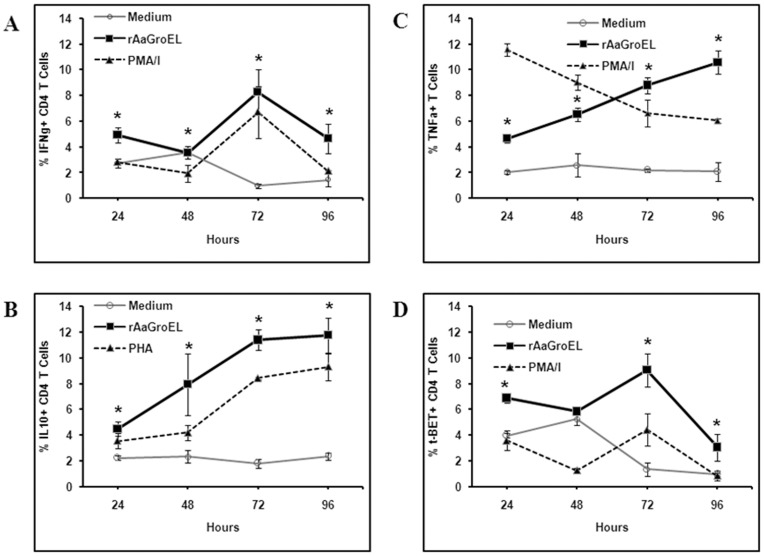
GroEL responding CD4+ T cells express IFNγ, IL-10, TNFα and T-bet. PBMCs cultured from 24 h to 96 h with rAaGroEL (20 µg/ml). RPMI and PMA/I or PMA were used as negative and positive controls respectively. At indicated time points CD4 cell surface staining was performed. Then, cytokine antibodies (IFNγ, IL10, TNFα and T-bet) were added. Cells were acquired and analyzed. For each molecule analysis, cells were gated for CD4+ T cells. *(A)*, *(B)*, *(C)*, *(D)* shows time kinetics of IFNγ, IL-10, TNFα and T-bet expression among CD4+ T cells with controls. Data are representative of 3 experiments. Error bars represent standard deviation and *represents p<0.05.

### Intracellular Cytokine or Transcription Factor Detection

The PMBC cultures were carried out as described above except for intracellular cytokine staining. To detect intracellular cytokines, 4 hours prior to the predetermined culture time, BD Golgistop™ (1∶1500) (BD Biosciences) was added to the cultures. At the end of the culture time, cells were first labeled with cell surface markers for T cells. Then cells were fixed and permeabilized. Following that antibodies for cytokines or transcription factors were added to cells. Labeled cells were analyzed by Flow cytometry.

### Statistical Analysis

The samples acquired by FACSArray and data were analyzed by FACSArray system software, FlowJo and FCAP softwares. Flow data were exported to MS Office Excel for further analysis. A two-tailed student’s t test was used for statistical analysis and p<0.05 accepted as statistically significant.

**Figure 4:**
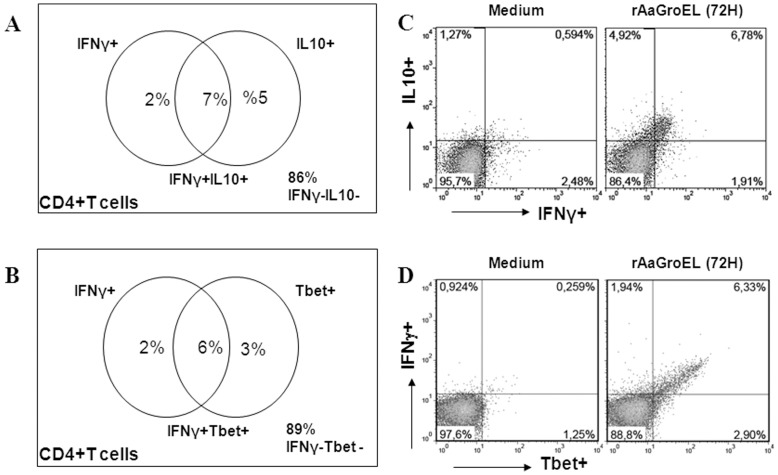
GroEL responding CD4+ T cells double cytokine producers. PBMCs cultured from 24 h to 96 h with rAaGroEL (20 µg/ml). RPMI and PMA/I were used as negative and positive controls respectively. At indicated time points CD4 cell surface staining was performed. Then, IFNγ, IL10 and T-bet antibodies were added to fixed and permed cells. Cells were acquired and analyzed. For each molecule analysis, cells were gated for CD4+ T cells. *(A*) shows representative distribution of IFNγ+IL-10+ double positive T cells, *(B)* shows representative distribution IFNγ+T-bet+ double positive T cells. *(C)* and *(D)* show representative flow data of double cytokine producing CD4+ T cells.

## Results

### rAaGroEL Protein Activates Human CD4+ T Lymphocytes

The antigenic stimulation of T lymphocytes induces expression of CD69, the earliest activation marker [10]. In order to investigate the antigenic effect of rAaGroEL on T lymphocytes, the CD69 expression was monitored on CD3+ T cells. We first cloned and over-expressed 64-kDa AaGroEL protein in *E.coli*. GroEL protein was purified (Fig. 1A) and the identity of protein was confirmed by Western blot (Fig. 1B). PBMCs were cultured with rAaGroEL at different concentration (1–40 µg) and at different time points. At 48 h, the CD69 expression was increased in a dose dependent manner and its expression on CD3+ T cells was highest when rAaGroEL was used at 20 µg/ml (Fig. 1C–D). Thus, 20 µg/ml concentration of rAaGroEL was determined as the optimum dose for activation of T lymphocytes and used for all subsequent screenings.

To identify whether rAaGroEL preferentially targets CD4+ or CD8+ T cells, rAaGroEL stimulated cells were analyzed simultaneously for CD69 and CD25 (IL-2R) expression [11] on the surface of CD4+ and CD8+ T cells. The CD4+ T cells expressed high level of CD69 molecules compared to CD8+ T cells (Fig. 2A). CD25 expression was increased CD4+ T cells in a time dependent manner but there was no detectable change in CD8+ T cells (Fig. 2B). Figure 2C shows representative distribution of PBMCs (percentage of CD14+, CD8+, CD4+ and other cells) and CD69 and CD25 expressing rAaGroEL responding CD4+ T cells. Data suggested that CD4+ T cells significantly increased both CD25 and CD69 expression (Fig. 2A–C), suggesting preferential targeting of CD4+ T cells by rAaGroEL. Thus, monitoring of CD4+ T cells response was pursued in greater details.

**Figure 5:**
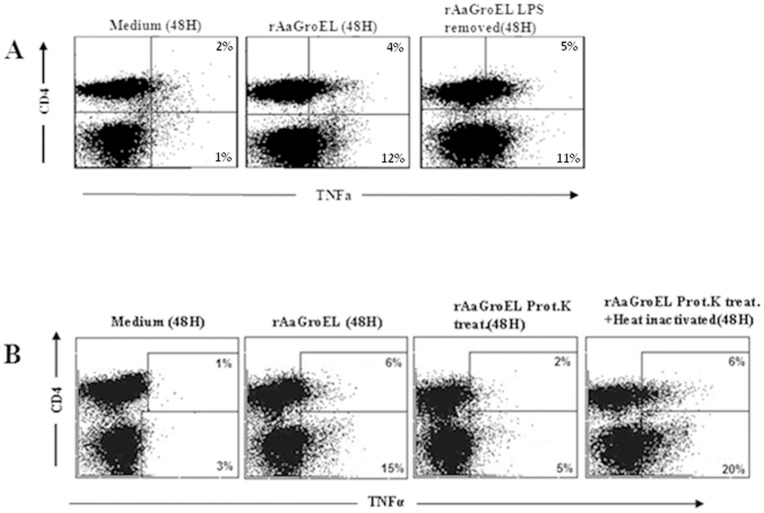
GroEL activity on CD4+ T cells is not due to LPS contamination. In order to test if the function came from possible LPS contamination, PMBCs were cultured with various antigens. *(A)* PBMCs were stimulated with rAaGroEL (20 µg/ml), LPS-removed-rAaGroEL (20 µg/ml; LPS was removed using Poly B supported resin). After 48 h stimulation cells were first labeled with CD4 antibodies. The cells, then, were fixed and permeabilized and incubated with anti-TNFα. Representative flow data shows TNFα expression among CD4+ T and non-CD4T cells. *(B)* PBMCs were incubated with rAaGroEL, Proteinase K-treated-rAaGroEL, heat-inactivated-Proteinase-K-treated-rAaGroEL and medium alone for 48 h and TNFα expression was measured. Representative flow data shows TNFα expression among CD4+ T and non-CD4T cells.

### rAaGroEL Responding CD4+ T Cells Express IFNγ, IL-10, TNFα and T-bet

Human CD4+ T cells can differentiate into at least four major subsets, Th1 [12], Th2 [13], Th9 [14] and Th17 [15] cells. After detecting the activated CD4+ T cells in the rAaGroEL-stimulated cultures, the phenotype of cytokine producing cells was investigated to determine which T cell subset differentiation is induced. Each Th subset produces their unique set of cytokines. IFNγ as Th1 phenotype major cytokine [13] was measured in rAaGroEL-stimulated cultures. Data showed that at 48 h, IFNγ expression reached up to 10% (p<0.05) and the level of IFNγ stayed elevated at 9.5% (p<0.05) at 96 h in rAaGroEL-stimulated cells (Fig. 3A). IL-10 was first discovered in Th2 T-cells that inhibits cytokine production [16] but it has recently been shown to be produced by many cells including CD4+ T cells [17]. We next investigated whether rAaGroEL-responding cells expressed IL-10. IL-10-expressing CD4+ T cells increased up to 12% in rAaGroEL-stimulated cells compared to 3% (p<0.05) (4-fold increase) in negative controls at 96 h (Fig. 3B). TNFα is an important pro-inflammatory cytokine and secreted by immune cells including T cells [18]. Whether rAaGroEL-responding CD4+ T cells express TNFα was also investigated. TNFα expressing CD4+ T cells in rAaGroEL-stimulated cultures increased from 2% up to 11% (5.5-fold increase) at 96 h compared to negative controls (Fig. 3C). Detecting IFNγ and IL-10 cytokines producing CD4+ T cells suggest that AaGroEL may commit CD4+ T cells into Th1 phenotype. It was previously reported that T-bet is a transcription factor that plays a role in Th1 lineage commitment by activating IFN-γ [19]. Thus, we hypothesized that if rAaGroEL-responsive CD4+ T cells are polarized into Th1 subset, they should be expressing T-bet. Data showed that T-bet expression continuously increased and reached 9% (Fig. 3D) compare to 0.2% in negative control cultures at 72 h.

### rAaGroEL Responding T Cells Concurrently Express IFNγ and IL-10

IFNγ is a major product of Th1 cells. Interestingly, many IFNγ-producing Th1 cells were found to produce IL-10 which is an anti-inflammatory cytokine in antimicrobial immune responses [20], [21]. To further investigate whether IL-10 and IFNγ cytokines were concurrently expressed by the same CD4+ T cells, IL-10 and IFNγ cytokines were measured at the same time. Data showed that IL-10 IFNγ expressing CD4+ T cells gradually increased up to 7% at 72 h in rAaGroEL-stimulated cultures. rAaGroEL responding CD4+ T cells were IL-10 and IFNγ double cytokine producing T cells (Fig. 4A). Additionally, 6% of IFNγ-expressing CD4+ T cells also expressed T-bet transcription factor at 72 h (Fig. 4B). Figure 4C–D shows representative flow data of double cytokine producing CD4+ T cells.

### rAaGroEL Activity is not due to LPS Contamination

The recombinant AaGroEL protein used in this study was expressed in *E.coli.* Therefore, the possibility that T cell activation or cytokine stimulating activity of rAaGroEL was due to LPS contamination was addressed. To that extent, we monitored TNFα expression in different culture condition, because rAaGroEL protein induced continuous expression of TNFα (Fig. 3C). Firstly, rAaGroEL was obtained from crude extract and LPS was removed from the purified rAaGroEL samples by using Poly B supported resin. Then, cells were cultured with purified rAaGroEL, LPS-removed-rAaGroEL and TNFα was measured among CD4+ T cells. Data showed that rAaGroEL-stimulated cells increased TNFα expression among CD4+ T cells and non-CD4 lymphocytes compared to negative controls. There were no significant differences in TNFα expression in purified-rAaGroEL or LPS-removed purified- rAaGroEL-stimulated cells (Fig. 5A). Additionally, purified-rAaGroEL treated with Proteinase K or heat-inactivated-proteinase K-treated-rAaGroEL- samples were cultured with cells. Results showed that there was a loss of TNFα stimulating function in Proteinase K-treated-rAaGroEL stimulated cells as compared to only rAaGroEL-stimulated cells (Fig. 5B). Altogether, these findings suggest that TNFα expression among CD4+ T cells was not due to LPS contamination (Fig. 4A and B).

## Discussion

The interest in the antigenic properties of bacterial Hsps comes from their ability to affect human host cells. It is known that different bacterial Hsps can induce inflammatory responses [22]. As a bacterial Hsp *A. actinomycetemcomitans* GroEL protein’s effect on immune system cells has not been studied yet. In this work, differentiation capacities of recombinant *A. actinomycetemcomitans* GroEL protein on human T lymphocytes were investigated. AaGroEL was cloned, expressed and purified. Then, recombinant AaGroEL was used as antigen source. To our best knowledge presented data demonstrated for the first time that rAaGroEL polarizes peripheral blood CD4+ T cells into an IFNγ IL-10 double cytokine producing T-bet+ Th1 phenotype.

Firstly, we showed that rAaGroEL-responding CD4+ T cells more successfully upregulate their CD69 and CD25 expression compared to CD8+T cells, suggesting that rAaGroEL could activate CD4+ T cells more effectively than CD8+T. Nevertheless, *H. pylori* hsp60 (also called HspB)-stimulated PBMCs showed proliferative responses but expression of activation markers IL-2R and HLA-DR on CD4+ and CD8+T cells were the same in infected and uninfected individuals [3]. Our finding suggests that different bacterial Hsp60s might affect T cells differently.

*Brucella abortus* GroEL heat-shock gene induces Th1 immune response. However, in this study soluble IFNγ and IL-4 cytokines were measured in mouse heterogeneous splenocytes but not IL-10 [5]. Additionally, Oliveira et al. showed that recombinant *Brucella abortus* GroEL protein primed T cells’ cytokine profile was characteristic of a Th1 type based on the detection of IL-2 and IFNγ [4]. Furthermore, recombinant GroEL of S*almonella typhi* immunized mice shifted the immune response toward Th1 phenotype where higher levels of IFNγ and IL-2 were detected [6]. However, these studies did not measure the intracellular cytokine expression with CD4 cell surface associated phenotypic marker, meaning the origin of cytokines is not well characterized. Thus, it is important to unravel the T cells that specifically secret these cytokines. Furthermore, none of these studies show GroEL responding CD4+ T cells can be double cytokine producing Th1 cells. It is important to point out presented study showed that recombinant GroEL protein of *A. actinomycetemcomitans* mediates IFNγ IL-10 double cytokine producing Th1 phenotype from CD4+ T cells.

Professional antigen presenting cells (APCs), appropriate TCRs for antigens, costimulatory and cytokine signals are required for CD4+ T cells to become activate. When T cells can become activated, they can proliferate and differentiate into different T helper (Th) subsets. Previously reported Th subsets are Th1 [12], Th2 [13], Th9 [14] and Th17 [15]. Th cells are named by their unique cytokine expression and carry out distinct effector functions. For instance, IFNγ is produced mainly by NK cells and T cells including Th1 cells. IL-10 was first discovered in Th2 cells. It was found that IL-10 is also produced by human Th1 T cells [17]. To investigate possible outcome of CD4+ T cell activation by rAaGroEL, whether CD4+ T cells differentiate into Th1 or not, phenotype specific cytokines were concurrently measured with CD4 cell surface marker on same T cells. Data showed that rAaGroEL-stimulated CD4+ T lymphocytes expressed significant level of IFNγ (Fig. 3A) and IFNγ-expressing CD4+ T cells were also expressing T-bet transcription factor (Fig. 4B and D). Morever, rAaGroEL-responding CD4+ T cells gradually increased their IL-10 expression (Fig. 3B). More importantly, IFNγ and IL-10 cytokines were simultaneously expressed on same CD4+ T cells in response to rAaGroEL (Fig. 4A and C). Taken together, the results obtained from this study elucidated that rAaGroEL-responding CD4+ T cells differentiated into IFNγ IL-10 expressing Tbet+ Th1 effector T cells.

IFNγ and IL-10 producing CD4+ T cell clones were first obtained in active tuberculosis patients [23] and patients with Lyme disease [24]. It is thus interesting that recombinant AaGroEL protein-mediated CD4+ T immune response polarized into IFNγ and IL-10 double cytokine producing Tbet+ Th1 cells. It was recently demonstrated that IFNγ IL-10 producing Th1 cells possess regulatory and effector functions. Th1 cells themselves are source of IL-10 that controls immune responses. Th1 effector responses are regulated through a negative feedback loop via the co-induction of IL-10 in addition to IFNγ in the same cells suggesting self control mechanisms of Th1 cells [25].

In this study, it was shown for first time that rAaGroEL-responding- CD4+ T were IL-10 and IFNγ double cytokine producing cells. These cells were also Tbet+. We concluded that rAaGroEL-responding CD4+ T cells differentiated into IL-10 IFNγ-expressing Tbet+ Th1 cells. Thus, an immune regulatory effect of recombinant AaGroEL on human T cells shown in this study would advance our understanding of *Aggregatibacter actinomycetemcomitans*-GroEL mediated immune response in inflammatory diseases.
